# Porcine Reproductive and Respiratory Syndrome Virus Evades Antiviral Innate Immunity *via* MicroRNAs Regulation

**DOI:** 10.3389/fmicb.2021.804264

**Published:** 2021-12-15

**Authors:** Xuan Zhang, Wen-Hai Feng

**Affiliations:** State Key Laboratory of Agrobiotechnology, Ministry of Agriculture Key Laboratory of Soil Microbiology, Department of Microbiology and Immunology, College of Biological Sciences, China Agricultural University, Beijing, China

**Keywords:** PRRSV, innate immunity, type I interferon, microRNA, immune evasion

## Abstract

Porcine reproductive and respiratory syndrome (PRRS) is one of the most important diseases in pigs, leading to significant economic losses in the swine industry worldwide. MicroRNAs (miRNAs) are small single-stranded non-coding RNAs involved in regulating gene expressions at the post-transcriptional levels. A variety of host miRNAs are dysregulated and exploited by PRRSV to escape host antiviral surveillance and help virus infection. In addition, PRRSV might encode miRNAs. In this review, we will summarize current progress on how PRRSV utilizes miRNAs for immune evasions. Increasing knowledge of the role of miRNAs in immune evasion will improve our understanding of PRRSV pathogenesis and help us develop new treatments for PRRSV-associated diseases.

## Introduction

PRRS was reported simultaneously in North America (1987) and Western Europe (1989), causing heavy reproductive failures in sows and acute respiratory distresses in piglets (Lunney et al., 2016). Since then, PRRS has quickly spread to the rest of the world and led to significant economic losses in the swine industry worldwide. In 2006, highly pathogenic PRRS (HP-PRRS) caused by a new type of PRRSV variant (HP-PRRS virus) was reported in China, which is characterized by high fever, high morbidity, and high mortality (Tian et al., 2007). Meanwhile, identification of NADC30 (a new genotype 2 strain) in the US in 2008 preceded the isolation of NADC30-like strains (with mortality rates of 30–50%) in central China in 2013 (Lei et al., 2015).

The causative agent, porcine reproductive and respiratory syndrome virus (PRRSV), was identified in the early 1990s in the Netherlands and the United States (Wensvoort et al., 1991; Morrison et al., 1992). PRRSV is an enveloped single-stranded positive-sense RNA virus belonging to the genus *Arterivirus*, family *Arteriviridae*, order *Nidovirales* (Carstens, 2010). According to sequence analysis, PRRSV is divided into two major genotypes, PRRSV-1 (species Betaarterivirus suid 1) and PRRSV-2 (species Betaarterivirus suid 2; Walker et al., 2019). PRRSV-1 and PRRSV-2 share about 50–70% nucleotide sequence identity (Kappes and Faaberg, 2015). The genome size of PRRSV is about 15.4 kb with at least 11 open reading frames (ORFs), encoding 8 structural proteins and at least 16 non-structural proteins (Li et al., 2015). Unlike other members in the genus *Arterivirus* that exhibit relatively broad cell tropism (Zhang and Yoo, 2015), PRRSV infection is highly restricted to cells of the monocyte–macrophage lineage such as porcine alveolar macrophages (PAMs), the primary targets of PRRSV *in vivo* (Lunney et al., 2016). Since the identification and characterization of PRRSV, new virulent PRRSV strains have constantly evolved by circumventing host immunity, and consequently, new outbreaks caused by new variants may never end.

microRNAs (miRNAs) are a conserved class of small non-coding RNAs, which play key roles in regulating almost every important biological process in all multicellular eukaryotes, including cell growth, proliferation, differentiation, and development. microRNAs function as regulators of gene expressions at the post-transcription levels through perfect or imperfect complementarity with the seed sequences in the target messenger RNA (mRNA), resulting in mRNA translation repression or degradation (Jonas and Izaurralde, 2015). Emerging evidence has indicated that viruses, including Epstein–Barr virus (EBV), dengue virus (DENV), Japanese encephalitis virus (JEV), and PRRSV, can escape host immune response by modulating microRNAs (Xia et al., 2008; Wu et al., 2013; Pareek et al., 2014; Zhang et al., 2016). This review will focus on the interaction between the host innate immune system and PRRSV, describing how PRRSV utilizes microRNA to escape host antiviral responses and signaling pathways.

## Host Innate Immune Responses To Prrsv Infection

### Host Innate Immune System

The innate immune response is the rapid first line of host defense to virus infection. It includes physical barriers such as skin and mucous membranes, chemical barriers including antimicrobial peptides, pH, lipids, enzymes, and immune cells such as monocytes, macrophages, eosinophils, neutrophils, and natural killer (NK) cells. Following virus infection, activating the innate immune system is critical to preventing cells from viral invasion and replication. More importantly, innate immunity plays essential roles in initiating the strong adaptive immune response to fight against intracellular pathogens (Lunney et al., 2016).

Interferons (IFN) are essential components of antiviral innate immunity against invading viruses by inhibiting intracellular propagation and the intercellular transmission of the virus (Ivashkiv and Donlin, 2014). Virus infection induces the development of an antiviral state within infected cells, and then, due to the concomitant secretion of IFN, leads to the establishment of an antiviral state in nearby cells. Thus, the IFN signaling pathway has two important activities: first, its induction, and second, its impact upon binding to IFN receptors present on many cell types. Type I and type II interferons (IFN-I and IFN-II) often work together to activate the innate and adaptive immune responses, eliminating viral infections (Lew et al., 1990; Schroder et al., 2004; Garcia-Sastre and Biron, 2006; Ivashkiv and Donlin, 2014). Whereas IFN-I is produced ubiquitously by virus-infected cells, secretion of IFN-II is restricted to cells of the immune system (Valente et al., 1992; Liu, 2005). Recent studies have been shown that type III interferons (IFN-III) have IFN-I-like immune responses and biological activities, directly performing an antiviral immune response at epithelial surfaces in the early stages of viral infection (Kotenko et al., 2003; Hamana et al., 2017).

IFN-I is inducible in essentially all cell types by several innate immune signaling pathways. The first step in activating different signaling pathways is recognizing the viral infection by the host cell through conserved pattern recognition receptors (PRRs). These receptors detect and distinguish molecules called pathogen-associated molecular patterns (PAMPs) that are part of microorganisms but not the host cells (Goubau et al., 2013; Brubaker et al., 2015). RNA virus infection is sensed by the toll-like receptors (TLRs) and the cytosolic sensors. TLR3 and TLR7, alternative endosome-lysosome PRRs, recognize double-stranded RNA and single-stranded RNA, respectively, leading to the dimerization of receptors and recruitment of adaptor TRIF or MyD88 (Alexopoulou et al., 2001; Oshiumi et al., 2003; Diebold et al., 2004). The stimulation triggers the assembly of signaling complexes and initiation of signaling cascades, resulting in the activation of interferon regulatory factor 3 (IRF3) and IRF7 and the transcription factor nuclear factor-κB (NF-κB) to induce the transcription of IFN-I (De Nardo, 2015). The retinoic acid-inducible gene I (RIG-I) and melanoma differentiation-associated gene 5 (MDA5), two intracellular PRRs, act as sensors to detect viral RNAs in the cytoplasm (Yoneyama et al., 2004; Kato et al., 2006). Activated RIG-I and MDA5 recruit and bind to mitochondrial antiviral signaling protein (MAVS, also known as VISA, Cardif, and IPS-1). MAVS relays the signal to TANK-binding kinase 1 (TBK1) and IκB kinase-ε (IKKε), which activate IRF3 and IRF7, which together with the NF-κB induces the expression of IFN-I (Yoneyama et al., 2015; Rehwinkel and Gack, 2020).

Most IFN-II expression is not directly induced by invading pathogens but is a secondary consequence of the infection. IFN-II is produced in the early stages of infections by NK cells, stimulated by macrophage-derived cytokines, especially TNF-α and IL-12, and at later stages by activated T lymphocytes (Darwich et al., 2009; Matsushita et al., 2014).

IFN-III are expressed in response to many classes of viruses and a variety of TLR agonists, in fact, the same stimuli responsible for the expression of IFN-I (Ank et al., 2008). IFN-I and IFN-III are both demonstrated to be induced by transcriptional mechanisms involving IRFs and NF-κB (Onoguchi et al., 2007). Despite the same transcriptional factors joining in the generation of IFN-I and IFN-III, the NF-κB pathway is the pivotal regulator in IFN-III generation, and the IRFs pathway dominates IFN-I expression. Furthermore, since IFN-III is induced through independent actions of IRFs and NF-κB, IFN-III expression seems more flexible than IFN-I expression (Osterlund et al., 2007; Iversen et al., 2010).

Upon IFN binding to cell surface receptors, a signal is transmitted into the cell, leading to the expression of hundreds of interferon-stimulated genes (ISGs) that may have direct antiviral activity. Although IFN-I, IFN-II, and IFN-III bind to distinct receptors, they activate a common intracellular signaling pathway (Donnelly and Kotenko, 2010). IFN-I is secreted in autocrine and paracrine manners and binds to the IFN-I receptor complex (IFNAR1 and IFNAR2). IFN-III binds a different receptor, consisting of the ubiquitously expressed IL-10R2 chain (shared with the IL-10 receptor) and a unique IFN-III receptor subunit (IFNLR1) that is restricted to epithelial cells (Kotenko et al., 2003; Donnelly et al., 2004). Both IFN-I and IFN-III activate the intracellular Janus kinase (JAK1 and TYK2), leading to the phosphorylation of both signal transducer and activator of transcription 1 (STAT1) and STAT2 (Schreiber and Piehler, 2015; Villarino et al., 2017). Upon phosphorylation, STAT1 and STAT2 form a heterodimer, which then combines with DNA-binding component interferon regulatory factor 9 (IRF9) to form a transcriptional complex, referred to as interferon-stimulated gene factor 3 (ISGF3; Fu et al., 1990). ISGF3 translocates into the nucleus to bind interferon-stimulated response elements (ISRE) and thereby drives the expression of hundreds of ISGs (Schneider et al., 2014). IFN-II, on the other hand, forms a homodimer and initiates the signal by interacting with two IFN-γ receptors 1 (IFNGR1) subunits, which leads to the binding of two additional IFNGR2 subunits and results in receptor activation (Walter et al., 1995). This activation leads to the phosphorylation of STAT1, which forms a homodimer. Then, the homodimer translocates into the nucleus and binds to gamma-activated sequence (GAS) promoter elements of ISGs (Decker et al., 1991, 1997). Thus, the induction of an antiviral state is achieved by the rapid and efficient activation of the JAK–STAT pathways, leading to the expression of multiple proteins encoded by ISGs, limiting virus replication and its subsequent spread to neighboring cells.

### PRRSV Strategies to Escape Host Innate Immune Response

During evolution, the continuous interaction between viruses and their hosts has shaped and determined their survival strategies. To replicate and spread in hosts, viruses have evolved multiple immune evasion mechanisms that result in the subversion of various cellular immune signaling pathways, particularly the inhibition of IFN. Unsurprisingly, downregulation of the IFN system, a powerful and first line of defense against virus infection, is a priority for most viruses. There are many strategies for viruses to antagonize the IFN system, including inhibition of IFN production, inhibition of IFN-mediated signaling pathways, and blocking the action of IFN-induced proteins with antiviral activity.

Generally, the IFN response is meager in PRRSV-infected pigs (Albina et al., 1998b) and remains low shortly after transient elevation below the detection level in the lungs of pigs where PRRSV actively replicates (Van Reeth et al., 1999). These studies have implied that the IFN response against PRRSV is poor, suggesting that the virus may actively suppress IFN production *in vivo*. Similarly, the suppression of IFN has also been observed in PRRSV infected Marc-145 cells and PAMs (Buddaert et al., 1998; Albina et al., 1998b; Miller et al., 2005). PRRSV still blocks the production of IFN-α when the cells are superinfected with swine transmissible gastroenteritis virus, a good IFN-α inducer (Albina et al., 1998a; Calzada-Nova et al., 2011). Moreover, PRRSV replication significantly inhibits the ds-RNA-induced IFN-I expression in Marc-145 cells (Miller et al., 2005). The poor induction of IFN-I response during infection suggests that PRRSV may have adopted strategies to suppress the induction and function of IFN-I. PRRSV proteins that are found to be antagonists of IFN induction can interact with multiple components of host IFN signaling pathways, thereby increasing the range and efficiency of their host evasion mechanisms. At least six viral proteins (nsp1α, nsp1β, nsp2, nsp4, nsp11, and N) have been identified as IFN antagonists, and their mechanisms of action are partially characterized. Nsp1α inhibits the association of IRF3 and CREB binding protein (CBP), enhances CBP degradation, and interferes with IκB degradation (Kim et al., 2010). Nsp1β inhibits the phosphorylation and nuclear translocation of IRF3 and degrades KPNA1 to block ISGF3 nuclear translocation (Wang et al., 2013b). Nsp2 inhibits IRF3 phosphorylation, interferes with IκB polyubiquitination, prevents IκB degradation, and suppresses the ISG15 pathway (Li et al., 2010; Sun et al., 2010, 2012). Nsp4 cleaves MAVS (VISA) and IKKγ (NEMO; Huang et al., 2014, 2016). Nsp11 degrades MAVS mRNA, induces STAT2 degradation to interfere with the formation of ISGF3, and inhibits nuclear translocation of ISGF3 (Sun et al., 2016; Yang et al., 2019). PRRSV N protein inhibits IRF3 phosphorylation and nuclear translocation and interferes with TRIM25-mediated RIG-I ubiquitination (Sagong and Lee, 2011; Zhao et al., 2019). Thus, PRRSV modulates the host antiviral immunity through the dysregulation of IFN production and function to establish infection in pigs.

More research has been emerging on the role of miRNAs in PRRSV strategies to escape host cell surveillance. Compared with proteins and long transcripts encoded by viral genes, miRNAs are more nonimmunogenic and flexible to restrain host cell defenses specifically and further establish a conducive cellular environment to the virus.

## Mirnas and Viral Infections

Since the discovery of the founding member of the miRNA family, *lin-4*, in the early 1990s (Wightman et al., 1993), thousands of miRNAs have been identified across the plants, animals, and viruses by molecular cloning and bioinformatic approaches (Berezikov et al., 2006; Ana et al., 2018). The process of miRNA biogenesis is highly complex. In the nucleus, most miRNA genes are transcribed by RNA polymerase II into primary transcripts of miRNAs (pri-miRNAs) that contain 5′caps and 3′poly (A) tails (Borchert et al., 2006). Pri-miRNAs are further processed to precursor miRNA (pre-miRNA) by the microprocessor complex consisting of the double-stranded RNA-binding protein DiGeorge syndrome critical region gene 8 and the RNase III endonuclease Drosha (Lee et al., 2003). The resulting pre-miRNAs are transported to the cytoplasm, a process mediated by exportin 5 in the presence of the co-factor Ran-guanine triphosphatase (Bohnsack et al., 2004). In the cytoplasm, the RNase III-type enzyme Dicer processes the pre-miRNA to a miRNA duplex, 20–25 nucleotides long from the stem of the pre-miRNA (Ketting et al., 2001). Finally, the miRNA duplex is handed over to a member of the Argonaute (AGO) protein family to assemble an RNA-induced silencing complex (RISC), which selects one strand to become the mature miRNA and discards the other strand (passenger strand; Gregory et al., 2005). Once incorporated into RISC, miRNA guides the complex to its mRNA targets by base-pairing interactions. In perfect or imperfect complementarity to the miRNA, target mRNAs can be cleaved (sliced) and degraded. Otherwise, their translation is repressed (Martinez and Tuschl, 2004). More recently, miRNAs have been shown to affect numerous processes of viral infections. Viruses manipulate the levels of particular miRNAs within the cell to establish a proviral environment that enhances viral replication and dissemination within and between hosts. On the other hand, viruses encode viral miRNAs to modulate host cell response to promote immune escape.

## Prrsv Immune Evasion *Via* Mirnas

### PRRSV-Mediated Changes in miRNAs to Escapes Immune Response

**Figure 1:**
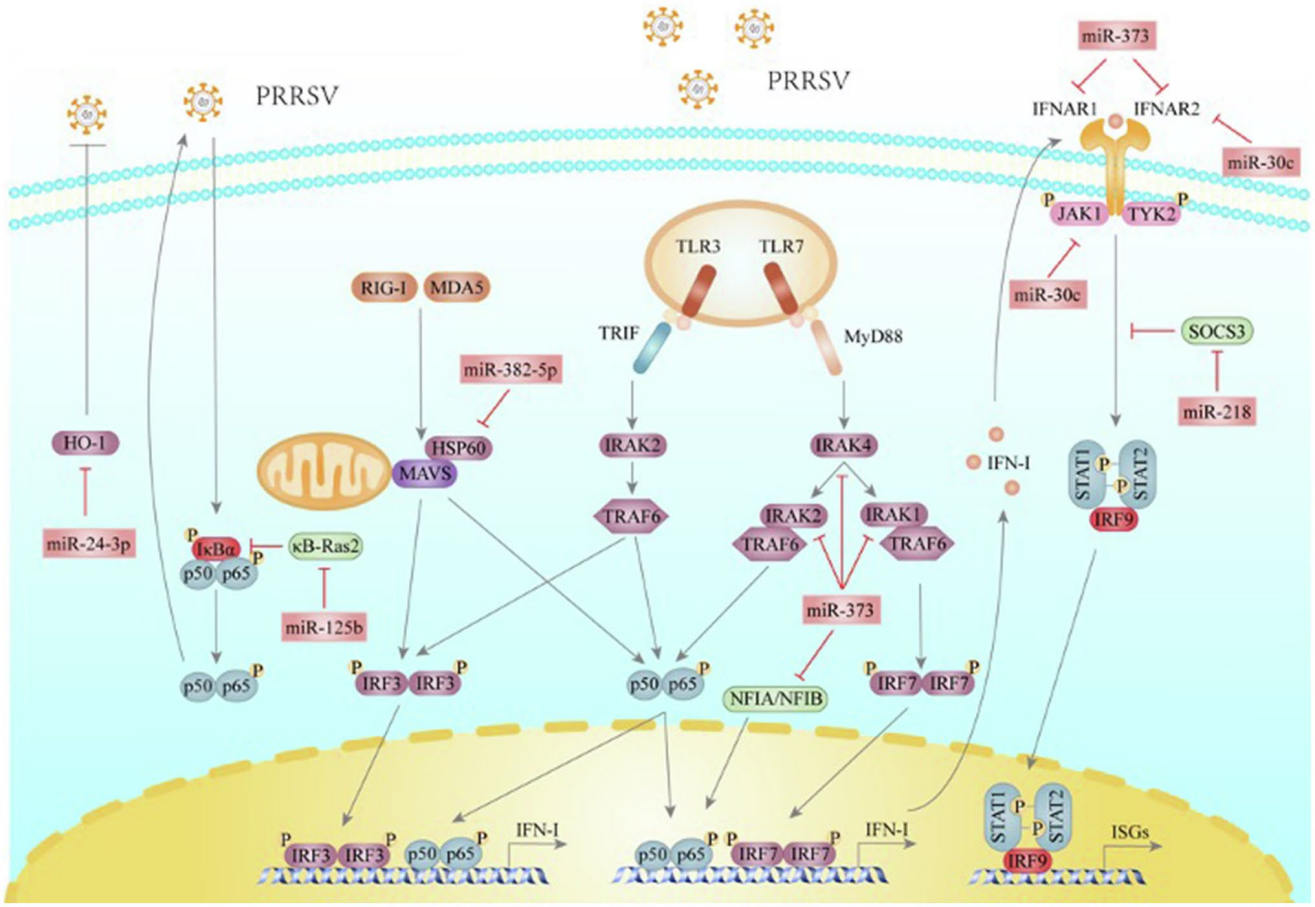
PRRSV utilizes miRNAs to help itself escape the host innate immune response. RIG-I and MDA5 detect dsRNA and initiate the adaptor protein MAVS to trigger IRF3 and NF-κB activations. TLR3, located at endosomes, senses dsRNA and transduces signals through TRIF, IRAK2, and TRAF6, leading to the activation of IRF3 and NF-κB. TLR7 senses ssRNA and transduces signals through MyD88, IRAK4, IRAK1, IRAK2, and TRAF6, subsequently activating IRF7 and NF-κB. After transferring to the nucleus, IRFs and NF-κB will transactivate the promoters to induce IFN-I expressions. IFN-I binds to IFNAR1 and IFNAR2 heterodimers, which transduce signals through recruiting JAK1 and TYK2, and result in STAT1 and STAT2 activation and binding to IRF9, constituting the ISGF3 complex. The complex translocates into the nucleus and promotes ISGs gene expression. SOCS3, a JAKs kinase inhibitor, can directly bind JAK1, JAK2, and TYK2 to inhibit their kinase activity and suppress IFN-mediated antiviral and anti-proliferative activities. κB-Ras2 binds directly to IκBα and inhibits IKK-dependent phosphorylation and subsequent degradation, thus inhibiting NF-κB signaling. HO-1 is an inducible enzyme and plays a critical role in immunomodulatory functions. NFIA and NFIB can induce interferon-β production. miR-373 is a novel negative miRNA to IFN-I signaling pathway by targeting NFIA, NFIB, IRAK1, IRAK2, IRAK4, IFNAR1, and IFNAR2 to promote PRRSV replication. miR-382-5p inhibits IFN-I production by targeting HSP60 and promotes PRRSV infection. miR-30c targets JAK1 and IFNAR2 to impair IFN-I signaling and then enhance PRRSV replication. PRRSV infection significantly down-regulates the expression of miR-218 to increase the expression of SOCS3, which inhibits the IFN-I response. PRRSV infection gradually decreases the expression of miR-125b, which relieves the stabilizing effect of SOCS3 on κB-Ras2, leading to NF-κB activation to facilitate PRRSV multiplication. miR-24-3p suppresses HO-1 expression to facilitate PRRSV replication and infection.

PRRSV infection influences various miRNAs expression in host cells (Hicks et al., 2013; Calcatera et al., 2018; Zhang et al., 2019). And then, cellular miRNAs could be used by PRRSV to reshape the cellular gene expression environment to the benefit of the virus. As reviewed below, we will discuss the miRNAs used by PRRSV to modulate host immune response to promote its infection (Figure 1).

#### miR-373

miR-373 is a multifunctional miRNA. It has been well documented that deregulation of miR-373 is involved in many cancers, and miR-373 can act as an oncogene or a tumor suppressor in different cancers (Wei et al., 2015). A recent study shows that PRRSV regulates the expression of host miR-373, which leads to immune escape and promotes virus replication (Chen et al., 2017). In the paper, the authors demonstrate that miR-373 promotes PRRSV replication since miR-373 is a negative regulator of IFN-β production by targeting interleukin-1 receptor-associated kinase 1 (IRAK1), IRAK4, and interferon regulatory factor 1 (IRF1). It is well known that IRAK1, IRAK4, and IRF1 are involved in the induction of IFN-I. After PRRSV infection, TLR3, TLR7, and RIG-I could utilize the adaptor molecules TRIF, MyD88, or MAVS to induce the production of IFN-I *via* IRAK1, IRAK4, TBK1, IRF1, IRF3, and IRF7 (Bowie and Unterholzner, 2008). Chen et al. (2017) find that miR-373 targets nuclear factor IA (NFIA) and NFIB (Chen et al., 2017). Both NFIA and NFIB could interfere with PRRSV replication by inducing the production of IFN-β, implying that NFIA and NFIB are novel antiviral proteins against PRRSV. In addition, miR-373 also targets IFNAR1 and IFNAR2, leading to the suppression of ISGs expression. Finally, the authors show that PRRSV upregulates the expression of miR-373 by elevating the expression of specificity protein 1 (Sp1). Thus, they conclude that PRRSV upregulates the expression of miR-373 by elevating the expression of Sp1 and then hijacks miR-373 to promote its replication by negatively regulating the production and function of IFN-β.

#### miR-382-5p

miR-382-5p is the primary miRNA species of miR-382. Previous studies have indicated that miR-382-5p plays an important role in tumor progression and acts as an oncogenic miRNA in several tumors, including breast cancer, liver cancer, acute promyelocytic leukemia, and glioma (Sun et al., 2019). Additionally, one report has investigated its relationship with virus replication and found that miR-382 expression is positively correlated with the viral load of human immunodeficiency virus-1 (HIV) among long-term non-progressors (Dey et al., 2016). The present research by Chang et al. shows that PRRSV infection upregulates the expression of miR-382-5p, which inhibits the production of IFN-I by targeting heat shock protein 60 (HSP60), thus achieving this goal PRRSV immune escape (Chang et al., 2020). HSP60, also known as HSPD1, is a molecular chaperone classically described as a mitochondrial protein (Marino Gammazza et al., 2018), facilitating IRF3phosphorylation and dimerization and interacting with the activated IRF3 (Lin et al., 2014). Meanwhile, HSP60 interacts with MAVS, an important signal transduction protein that activates IRF3 and NF-κB, enhancing MAVS-mediated IFN-I signaling. Overexpression of HSP60 inhibits PRRSV replication in Marc-145 cells, and knockdown of HSP60 promotes PRRSV replication, indicating that HSP60 is an antiviral protein against PRRSV replication. Thus, PRRSV upregulates the expression of miR-382-5p, which negatively regulates the production of IFN-I through targeting HSP60, leading to the enhancement of PRRSV replication.

#### miR-30c

The miR-30 family is an important member of the miRNA family, containing five members (miR30a–e; Mao et al., 2018). Previous studies have suggested that the miR-30 family is involved in the regulation of immune response. The miR-30 targets Beclin1 and ATG6 to regulate autophagy (Yang and Pan, 2015; Zhang et al., 2015). miR-30b is shown to enhance IL-10 and NO production through targeting Notch1 (Su et al., 2013). Our study shows that PRRSV escapes IFN-I-mediated antiviral immune responses by engaging miR-30c (Zhang et al., 2016). Following pathogen detection and subsequent IFN production, IFN bind to cell surface receptors and initiates a signaling cascade through the JAK–STAT pathway, resulting in the transcriptional regulation of hundreds of ISGs and leading to a great antiviral state (Schneider et al., 2014). We show that PRRSV infection upregulates miR-30c primarily through the NF-kB signal-transduction pathway. Importantly, we have confirmed that miR-30c is also upregulated by PRRSV infection *in vivo*, and miR-30c expression positively correlates with the viral loads in lungs and PAMs of PRRSV-infected pigs. In addition, miR-30c also targets the IFN-I receptor IFNAR2 and suppresses the production of ISGs (Liu et al., 2018). Collectively, PRRSV infection significantly induces miR-30c expression in an NF-kB-dependent manner, which targets JAK1 and IFNAR2 to impair IFN-I signaling and enhance PRRSV replication.

#### miR-24-3p

miR-24-3p has emerged as a crucial miRNA and played important roles in several diseases. miR-24-3p is highly expressed in tumor tissues and promotes cell proliferation, migration, and invasion in cancer cells (Lin et al., 2018; Yan et al., 2018). Moreover, miR-24-3p negatively regulates inflammation in various diseases (Devadas and Dhawan, 2006; Shi et al., 2009; Paine et al., 2010; Pua et al., 2016; Jingjing et al., 2017). A study finds that PRRSV infection markedly upregulates miR-24-3p and positively regulates PRRSV replication in Marc-145 cells and PAMs (Xiao et al., 2015). miR-24-3p regulates heme oxygenase-1 (HO-1) expression, an inducible enzyme strongly induced by hemin and various stressors, including viral infection. HO-1 may reduce pro-oxidant levels and protect cells from death and apoptosis triggered by oxidative stress conditions (Hamedi-Asl et al., 2012). Indeed, increasing evidence suggests that host cell oxidative stress status plays an important role in viral replication and infectivity (Cai et al., 2003). Significantly, overexpression or induction of HO-1 greatly inhibits PRRSV replication. Thus, it is reasonable to conclude that PRRSV remarkedly upregulates miR-24-3p to facilitate its replication and infection (Xiao et al., 2015).

#### miR-218

miR-218 is first described in prostate cancer and gastric cancer and has been shown to serve as a tumor suppressor by inhibiting glioblastoma invasion and proliferation in different types of cancers (Tie et al., 2010; Leite et al., 2011). Recently, miR-218 has been shown to contribute to other processes, including apoptosis and inflammation response (Li et al., 2020; Wang et al., 2020). Recently, it has been reported that PRRSV enhances its replication by downregulating miR-218 (Zhang et al., 2021). PRRSV infection significantly reduces miR-218 expression. Subsequent research finds that miR-218 directly binds to suppressor of cytokine signaling 3 (SOCS3) mRNA to repress SOCS3 expression. It is well known that the JAK family plays crucial roles in mediating IFN-dependent biological responses, including activation of the antiviral state. SOCS3, a JAKs kinase inhibitor, can directly bind JAK1, JAK2, and TYK2 to inhibit their kinase activity from suppressing IFN -mediated antiviral and anti-proliferative activities (Zimmerer et al., 2006; Babon et al., 2009). Thus, miR-218 augments the antiviral activity of IFN-I by targeting SOCS3. In summary, PRRSV-induced miR-218 downregulation serves to inhibit the IFN-I response, which allows PRRSV to evade the protective innate immune response within host cells.

#### miR-125b

miR-125 family, consisting of miR-125a and miR-125b, is a highly conserved miRNA family (Wang et al., 2019). miR-125b has gained a special interest in virus research because of its roles in immunity. Present studies have indicated that miR-125b is highly expressed in peripheral blood mononuclear cells, and its expression is inversely associated with PRRSV infection (Wang et al., 2013a). Ectopically expressed miR-125b reduces PRRSV progeny production. Conversely, inhibition of miR-125b substantially enhances PRRSV propagation. miR-125b targets the 3′UTR of κB-Ras2. κB-Ras2 is a member of the Ras family of proteins that negatively regulate NF-κB signaling. κB-Ras2 binds directly to IκBα and inhibits IKK-dependent phosphorylation and subsequent degradation, thus inhibiting NF-κB signaling (Huxford and Ghosh, 2006). Therefore, miR-125b negatively regulates NF-κB by stabilizing κB-Ras2 mRNA. Although NF-κB has long been considered a key transcription factor for innate immune responses, PRRSV redirects NF-κB’s activity of NF-κB into a viral supportive function (Lee and Kleiboeker, 2005). PRRSV increases NF-kB activity at late phases of infection, and inhibition of NF-κB activation suppresses the production of progeny PRRSV virus (Wang et al., 2013a). PRRSV gradually decreases the expression of miR-125b, which, in turn, relieves the stabilizing effect on κB-Ras2, and ultimately leads to the activation of NF-κB. However, the elaborate mechanisms by which PRRSV regulates NF-kB activation and promotes PRRSV replication warrant further studies.

In addition to the above miRNAs, PRRSV infection regulates other miRNAs such as miR-146a, miR-29, and miR-147. miR-146a is one of the best-characterized vertebrate miRNAs and plays various roles in the innate immune system. Previous studies have demonstrated that enterovirus 71 (EV71), DENV, and JEV can induce miR-146a expression in infected cells. miR-146a negatively regulates IFN signaling through targeting tumor necrosis factor receptor-associated protein 6 (TRAF6) and IRAK1, thus preventing the induction of IFN-I from promoting viral infections (Siyu et al., 2013; Ho et al., 2014; Sharma et al., 2015). miR-146a is also upregulated in PRRSV-infected macrophages, suggesting that miR-146a is likely to play a role in PRRSV immune evasion (Hicks et al., 2013). the miR-29 family is shown to be key miRNAs in regulating adaptive immune response, suggesting that they might participate in viral infection (Kogure et al., 2012). Interestingly, both miR-29a and miR-29b are significantly upregulated in PAMs infected with PRRSV, and overexpression of miR-29a or miR-29b promotes PRRSV replication in cells. However, whether PRRSV upregulates miR-29a to inhibit innate immune responses remains to be investigated. miR-147 is down-regulated in PRRSV-infected macrophages, and transfection of miR-147 mimics results in a significant decrease in PRRSV replication (Hicks et al., 2013). These results imply that the decline of miR-147 levels upon PRRSV infection may benefit virus production.

As described above, PRRSV utilizes multiple host miRNAs to adapt to the host immune response and promote immune evasion. However, whether other miRNAs regulated by PRRSV enhance immune evasion remains to be further explored.

### PRRSV-Encoded miRNAs to Escapes Immune Response

RNA viruses may encode miRNAs. Nuclear RNA viruses, including retroviruses and orthomyxoviruses, complete their transcription and replication in host cells’ nucleus and are identified to encode miRNAs (Pfeffer et al., 2005; Harwig et al., 2014). Unlike nuclear RNA viruses, cytoplasmic RNA viruses that spend their whole life cycle in the cytoplasm without a DNA intermediate are considered the least likely to encode bona fide miRNAs because of both a lack of access to classical miRNA microprocessors in the nucleus and the threat of genome destabilization (Rouha et al., 2010; Shapiro et al., 2010). Nonetheless, many cytoplasmic RNA viruses have been verified to encode miRNAs to circumvent these potential barriers, including Ebola virus (EBOV), West Nile virus (WNV), and Dengue virus 2 (Mazhar et al., 2012; Hussain and Asgari, 2014; Liang et al., 2014). So far, more than 30 virus-encoded miRNAs have been identified from RNA viruses.

Recently, it has been reported that PRRSV encodes viral small miRNAs (vsRNAs). Four vsRNAs were mapped to the stem-loop structures in the ORF1a, ORF1b, and GP2a regions of the PRRSV genome (Wei, 2010). Of these, PRRSV-vsRNA1 stem-loop structures exhibit the stability of a typical hairpin structure. PRRSV-vsRNA1 has been verified to target the NSP2 region and partially prevent virus replication from avoiding virus over-replication and premature death of host cells to establish persistent infection (Na et al., 2016). Several targets of PRRSV vsRNAs related to the immune system are predicted, suggesting that PRRSV seems to exploit vsRNAs to inhibit specific host genes and escape the immunological surveillance and speed its genome replication (Wei, 2010). The discovery of these PRRSV vsRNAs adds more insights into how the PRRSV disturbs the host normal function of the immune system. However, pitfalls still exist in current studies. Also, controversies that cytoplasmic RNA viruses encode miRNAs still exist partly due to several barriers. For example, cytoplasmic RNA viruses have no access to the whole miRNA processing machinery, and viral genome instability is induced by endonuclease that can recognize the miRNA-encoding stem-loop structure (Cullen, 2010). Thus, results from these studies need a more well-established methodology combining multiple computational and experimental analyses to yield reliable and reproducible results.

## Concluding Remarks and Perspectives

Both viruses and host cells can manipulate miRNAs to regulate innate responses as part of their evolutionary strategies. PRRSV uses miRNAs to evade the host innate immune system and maintain its infection by reducing host antiviral gene expression. Further elucidating the roles of PRRSV-regulated and PRRSV-encoded miRNAs in fundamental processes associated with PRRSV immune escape will help us understand PRRSV pathogenesis. Also, considering the advanced stage of the development of Miravirsen, an oligonucleotide that has been shown to inhibit HCV infection by interfering with miR-122a function, miRNA-based therapeutic could be a promising strategy. Thus, our studies need to place more emphasis on identifying potential targets. At a stage where an effective anti-PRRSV therapy is still lacking, blocking miRNAs, which relate to PRRSV innate immune evasion, may serve as a straightforward approach to develop new antivirals.

## Author Contributions

XZ summarized current progresses on how PRRSV utilizes miRNAs for immune evasions and wrote this review. W-HF provided guidance and modified this review. All authors contributed to the article and approved the submitted version.

## Funding

The study was supported by the National Natural Science Foundation of China (Grant No. 31630076), China, and the National Major Special Project on New Varieties Cultivation for Transgenic Organisms (Grant No. 2016ZX08009-003-006), China.

## Conflict of Interest

The authors declare that the research was conducted in the absence of any commercial or financial relationships that could be construed as a potential conflict of interest.

## Publisher’s Note

All claims expressed in this article are solely those of the authors and do not necessarily represent those of their affiliated organizations, or those of the publisher, the editors and the reviewers. Any product that may be evaluated in this article, or claim that may be made by its manufacturer, is not guaranteed or endorsed by the publisher.
